# Sequences of acetyl CoA carboxylase promoter for tumour necrosis factor action

**DOI:** 10.1155/S0962935193000377

**Published:** 1993

**Authors:** Keerang Park, Michael E. Pape, Ki-Han Kim

**Affiliations:** Department of Biochemistry Purdue University West Lafayette IN 47907 USA

## Abstract

Tumour necrosis factor (TNF) inhibits the accumulation of acetyl CoA carboxylase (ACC) mRNA by decreasing the rate of ACC gene transcription. The ACC mRNA species found in 30A5 cells are generated from promoter II and TNF inhibits the accumulation of class 2 type mRNAs. By using 5' deletion mutants of promoter II fused to the bacterial chloramphenicol acetyltransferase (CAT) gene, the DNA mobility shift assay and the DNase I footprinting assay, the authors have identified the 30 bp from −389 to −359 as the TNF responsive element in promoter II. TNF treatment causes a decrease in the binding activity of nuclear protein(s) specific to the TNF responsive element. When the fragment containing the TNF responsive element was incorporated into the thymidine kinase promoter, the chimeric gene exhibited TNF induced inhibition of expression.


Mediators of Inflammation 2, 271–277 (1993)

### Sequences of acetyl CoA carboxylase promoter for tumour necrosis factor action

Keerang Park, Michael E. Pape and Ki-Han Kim[CA]

Department of Biochemistry, Purdue University, West Lafayette, IN 47907, USA

[CA] Corresponding Author

**TUMOUR necrosis factor (TNF) inhibits the accumulation of acetyl CoA carboxylase (ACC) mRNA by decreasing the rate of ACC gene transcription. The ACC mRNA species found in 30A5 cells are generated from promoter II and TNF inhibits the accumulation of class 2 type mRNAs. By using 5′ deletion mutants of promoter II fused to the bacterial chloramphenicol acetyltransferase (CAT) gene, the DNA mobility shift assay and the DNase I footprinting assay, the authors have identified the 30 bp from −389 to −359 as the TNF responsive element in promoter II. TNF treatment causes a decrease in the binding activity of nuclear protein(s) specific to the TNF responsive element. When the fragment containing the TNF responsive element was incorporated into the thymidine kinase promoter, the chimeric gene exhibited TNF induced inhibition of expression.**

**Key words:** Acetyl CoA, Gene transcription, mRNA, Tumour necrosis factor

### Introduction

The presence of a chronic infection or malignancy induces macrophages to produce TNF and subsequently drives bodily metabolism towards a constitutive catabolic state, the primary characteristic of cachexia. A hallmark of cachexia is the depletion of lipid reserves from adipocytes and the accumulation of serum lipids.[1] Oliff and co-workers have demonstrated that nude mice harbouring solid tumours which secrete TNF display 'cancer-associated cachexia'. These mice show a marked loss of intrascapular fat pads, perirenal and subcutaneous fat. Although it is difficult to directly assess the role of TNF in altering fat depots and inducing cachexia in this animal model,[2,3] it is apparent that TNF has a marked effect on lipid metabolism. This is also borne out in other systems.[4–7]

TNF completely inhibits the conversion of 3T3–L1, TA1 and 30A5 preadipocytes to adipocytes in *in vitro* culture systems.[4–7] In addition, TNF can also induce fully differentiated adipocytes to revert to a preadipocyte like state under certain culture conditions.[6–8] The aim of this work has been to understand how TNF alters lipid metabolism by studying the effect of the monokine on acetyl CoA carboxylase gene expression in 30A5 preadipocytes.

Acetyl CoA carboxylase (ACC) catalyzes the rate-limiting step in the biosynthesis of long-chain fatty acids.[9] It has been shown that TNF completely inhibits the conversion of 30A5 preadipocytes to adipocytes with the concomitant inhibition of the accumulation of ACC mRNA.[5,10] TNF suppresses ACC mRNA accumulation by decreasing the rate of transcription of the ACC gene.[10] ACC mRNA exists in multiple forms which are generated as a result of differential splicing of two primary transcripts of two distinct promoters, promoters I and II.[11,12] Expression of these promoters is tissue specific and dependent upon physiological conditions. In the case of 30A5 cells, the primary gene products are those mRNAs produced under the influence of promoter II, and there is little or no ACC mRNA from promoter I.[13] In this paper, the promoter II region of the ACC gene which confers TNF responsiveness to the gene has been characterized. The authors show that a 30 bp fragment in the 5′ flanking sequences of the ACC gene confers TNF mediated inhibition of transcription. In addition, evidence that specific binding of nuclear proteins to the 30 bp fragment is decreased upon treatment of 30A5 cells with TNF, is presented.

### Materials and Methods

*Materials:* The following commercial products were used: Eagle basal medium (MA Bioproducts, Walkersville, MD); foetal bovine serum (GIBCO Laboratories, Grand Island, NY); dexamethasone and insulin (Collaborative Research, Inc., Waltham, MA); T4 DNA polynucleotide kinase, restriction enzymes (New England Biolabs, Beverly, MA); DNA polymerase Klenow fragment, DNase I, exonuclease III, and calf intestinal alkaline phosphatase (Boehringer Mannheim, Indianapolis, IN); $^{14}C$-labelled chloramphenicol, [$\gamma$-$^{32}P$]-adenosine 5′-triphosphate (Amersham, IL); recombinant human

© 1993 Rapid Communications of Oxford Ltd

TNF-α ($2.7 \times 10^7$ U/mg) was generously provided by Dr Tatsuro Nishihara of the Suntory Institute, Osaka, Japan.

*Cell culture:* The 30A5 preadipocyte cells were grown in Eagle basal medium containing 10% foetal bovine serum or bovine calf serum (Hyclone, Logan, UT) as described previously.[14] TNF included in the medium at a concentration of 200 U/ml, from day 0 (the day of confluence, D–0) prevents differentiation under two differentiation schemes.[14] These cells are called TNF treated preadipocytes.

*Plasmid constructions:* Construction of the plasmids, pUC-CAT3 and plasmid pPII-CAT0 has been described previously.[12] The plasmid pPII-CAT0 contains the −994 to +62 (relative to the first transcription initiation start site) region of promoter II in a 5′ to 3′ orientation relative to the CAT gene. The exonuclease III method and standard recombinant DNA techniques[15] were used to generate a series of 5′ deletion mutants of ACC promoter II. The end point and orientation of the deletions were determined by DNA sequencing.[15]

Putative regulatory elements were placed into a thymidine kinase (TK)/CAT fusion gene. pTK–CAT[12] which contains the −109/+51 region of the TK promoter distal to the CAT gene was digested at the unique *Sal*I site and filled in. Various fragments of ACC promoter II, as indicated in the text, were blunt-end ligated into this site.

The synthesized mouse TNF responsive sequence spanning −370 to −340 was flanked by *Sac*I restriction sites and used for cloning. These oligonucleotides are as follows: 5′–GGTGGAGGTTGGCCGGCCCAAACCCGC-CTAGCT–3′, and 5′–AGGCGGGTTTGGGCCG-GCCAACCTCCACCAGCT–3′. The synthesized complementary strands were annealed, phosphorylated by T4 polynucleotide kinase and ligated into pPII–CAT4 plasmid vector which was digested with *Sac*I restriction enzyme and dephosphorylated with calf intestinal alkaline phosphatase. All constructed mutants were confirmed by DNA sequencing.[15] The features of all gene constructs used are shown in Fig. 1.

*Transfections and CAT assays:* Cell monolayers at 80–90% confluence were transfected with 20 μg of ACC–CAT construct DNA by coprecipitation with calcium phosphate.[16] Cells were exposed to the precipitate for 8 h at which time fresh medium with or without 200 U/ml of TNF was added. Cell lysates were made 60 h later by sonication in 150 μl of 0.25 M Tris-HCl, pH 8.0 followed by heating at 60°C for 10 min to minimize the effect of deacylating agents in the CAT assay.[17] The CAT assay was performed essentially as described.[16]

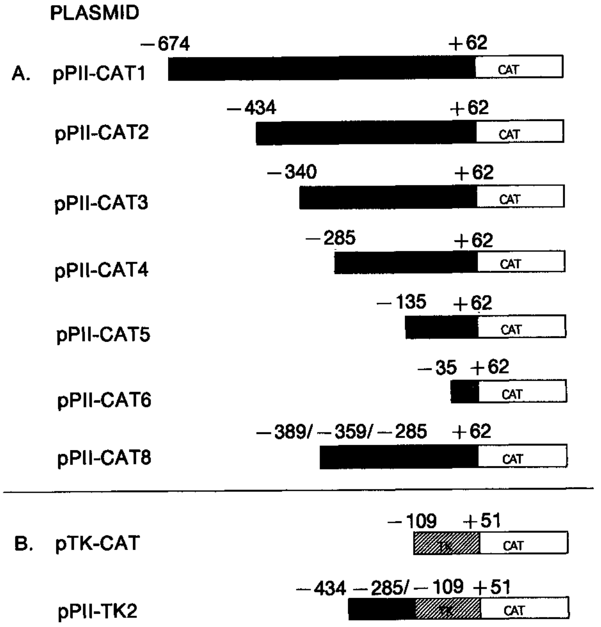

FIG. 1. Schematic diagram of pPII–CAT and pPII–TK constructs. (A) Acetyl CoA carboxylase promoter II fragments were inserted upstream of the CAT gene in pUC–CAT3. The numbers in the figures refer to the nucleotide positions in the promoter; the value +1 was assigned to the transcription initiation site. (B) The promoter fragment ranging from −434 to −285 was inserted in front of pTK–CAT to create pPII–TK2.

Protein amount was determined using the Bradford reagent with bovine serum albumin as the standard.[14] CAT activity was expressed as percentage chloramphenicol acetylated per h per mg protein. A beta-actin-CAT construct[18] was included in each experiment to correct for any differences in transfection efficiency.

*Preparation of nuclear extracts:* Nuclear extracts were prepared according to the method of Dignam *et al.*[19] 30A5 cells at D+4 which were fully differentiated by the cAMP pretreatment differentiation scheme,[14] or the cells at D+4 which were kept in TNF (200 U/ml) during the same differentiation scheme, were used for preparations. Nuclear extracts were divided into small aliquots, quickly frozen in liquid nitrogen and stored at −80°C.

*DNA mobility shift assays:* For oligonucleotides, both complementary single-stranded 30-mers (−389 to −359) were end-labelled separately. The labelled 30-mers were annealed in 140 mM KCl and purified by gel electrophoresis in a 20% polyacrylamide gel or by using a Chroma Spin + TE-10 column. The labelled DNA fragments were incubated with 10 μg of nuclear proteins on ice for 30 min, in 20 μl of binding buffer (5 mM $MgCl_2$, 5 mM DTT, 1 mM EDTA, 50 mM KCl, 10 mM HEPES, pH 7.9, 10% glycerol, and 1 μg poly (dI.dC). For competition experiments, different molar concentrations of non-labelled specific competitors or nonspecific competitors were added to the reactions. DNA

mobility retardation was analyzed by a 4% polyacrylamide gel electrophoresis, and autoradiography.

*DNase I footprinting:* The labelled probe for the DNase I footprinting analysis was pPII–TK2 which contains the ACC promoter II fragment from −434 to −285 in pTK–CAT vector using blunt-ended *Sal* I site. pPII–TK2 was digested with *Hind*III restriction enzyme, dephosphorylated with calf intestinal alkaline phosphatase, and radiolabelled by using [$\gamma$-$^{32}$P]ATP and T4 polynucleotide kinase. The labelling was followed by *Eco*RI digestion. The 224 bp labelled fragment containing the ACC promoter fragment (−434 to −285) and 34 bp of TK promoter sequences was isolated through electrophoresis from the gel following its separation. Five nanograms (20 000 cpm) of the labelled DNA fragment was incubated with 40 μg of nuclear extracts in a binding reaction (50 μl) at 0 °C for 30 min. The binding mixture was then subjected to DNase I (2 μl of DNase I solution containing different units of the enzyme as specified) treatment at room temperature for 2 min. The reaction was terminated by addition of 120 μl stop buffer (30 mM EDTA, 1% SDS, 300 mM NaCl and 250 μg of tRNA per ml). Samples were deproteinized by extraction with phenol–chloroform–isoamyl alcohol 25:24:1), precipitated with ethanol, and loaded onto an 8% denaturing polyacrylamide gel.

### Results

*Identification of* cis-*acting DNA elements mediating TNF responsiveness:* In 30A5 cells, ACC mRNA species are synthesized under the influence of promoter II (PII), one of the two promoters in the ACC gene, and no detectable ACC mRNA species are synthesized from promoter I.[13] TNF inhibits the rate of transcription of the ACC gene during the transformation of 30A5 preadipocyte to adipocytes.[10]

A series of 5′ deletion mutants in which deleted fragments of ACC promoter II were fused upstream of the CAT gene[12] was prepared as shown in Fig. 1A, and the effect of the promoter II fragment on CAT gene expression was assessed in a transient expression system (Fig. 2). The data indicate that there are several regions within the −674 to +62 sequences important in the expression of the ACC gene (Fig. 2). The −674 to −434 region appeared to contain a negative control element as CAT activity increased about three-fold when this sequence was deleted. However, the −434 to −135 region contained positive elements as evidenced by the decrease in CAT activity when these sequences were deleted. The most important positive element appeared to lie between −285 and −135, as a

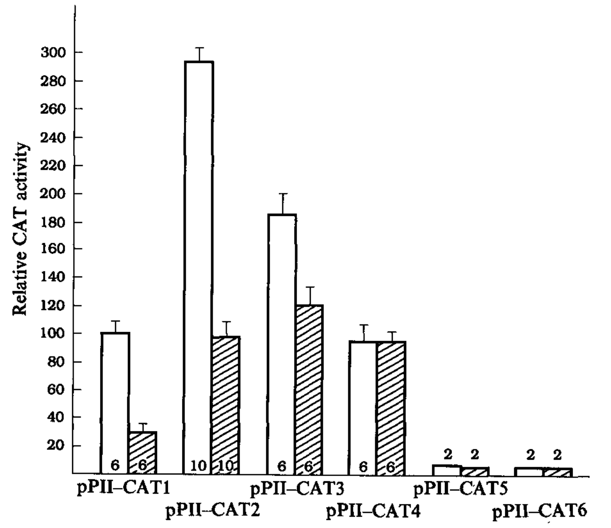

FIG. 2. Relative transcription rates of 5′ deletion mutants of acetyl CoA carboxylase promoter II. Various carboxylase promoter II/CAT constructs as shown in Fig. 1A were transfected into subconfluent 30A5 cells followed by a 60 h incubation in the absence or presence of TNF. The CAT activity obtained from pPII–CAT1 which was included in each experiment was set as 100%. The number of plates used to obtain data for each bar is indicated. Error bars indicate the range of three determinations. The size of PII in each construct is: pPII–CAT1, −674/+62; pPII–CAT2, −434/+62; pPII–CAT3, −340/+62; pPII–CAT4, −285/+62; pPII–CAT5, −135/+62; pPII–CAT6, −35/+62. □, Control; ▨ TNF.

greater than ten-fold decrease in CAT activity resulted when pPII–CAT4 and pPII–CAT5 were used. Recently, the authors reported the presence of a strong enhancer sequence in this region which is neither promoter specific nor orientation specific.[12]

To determine if TNF could suppress the expression of the CAT gene in these chimeric gene constructs, TNF was added to 30A5 preadipocytes after transfection with the various constructs. Figure 2 shows that the sequences responsible for TNF mediated inhibition of ACC gene transcription do indeed lie within the −674 to +62 region. Plasmids pPII–CAT1 and pPII–CAT2 showed about 70% suppression of CAT activity upon TNF treatment in comparison to untreated controls. However, pPII–CAT4, pPII–CAT5, and pPII–CAT6 did not show suppression by TNF, suggesting that the elements responsible for mediating the TNF effect may lie between −434 and −285. To further define the role of these sequences in mediating the TNF response, we inserted a fragment spanning the −434/−285 region upstream to the thymidine kinase promoter which was linked to the CAT gene (Fig. 1B). When placed distal to the thymidine kinase promoter in pTK–CAT, the 150 bp fragment conferred TNF responsiveness to the TK promoter (Fig. 3). TNF caused about a 65% suppression of CAT activity in pPII–TK2. Previously, the authors reported that TNF treatment of 30A5 cells decreased the rate of transcription of the ACC gene by 60 to 70%.[10] Thus,

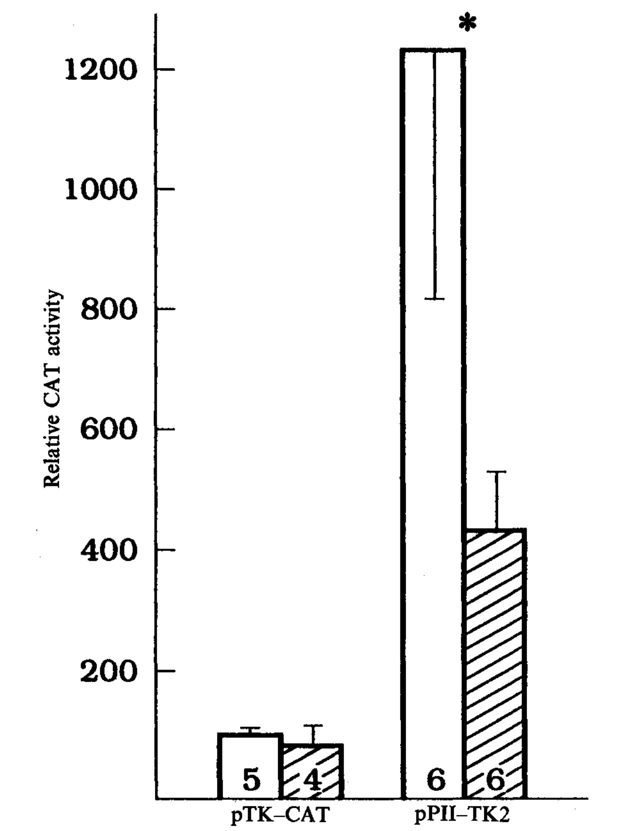

FIG. 3. Effect of acetyl CoA carboxylase promoter II sequences in a TK/CAT construct. The sequence spanning −434 to −285 was placed distal to the TK promoter in pTK–CAT to form pPII–TK2. The effect of TNF on CAT activity was assayed as described in Materials and Methods. The CAT activity obtained from pTK–CAT was set as 100%. The number of plates used to obtain data for each bar is indicated. Error bars indicate the range of three determinations. Key as Fig. 2.

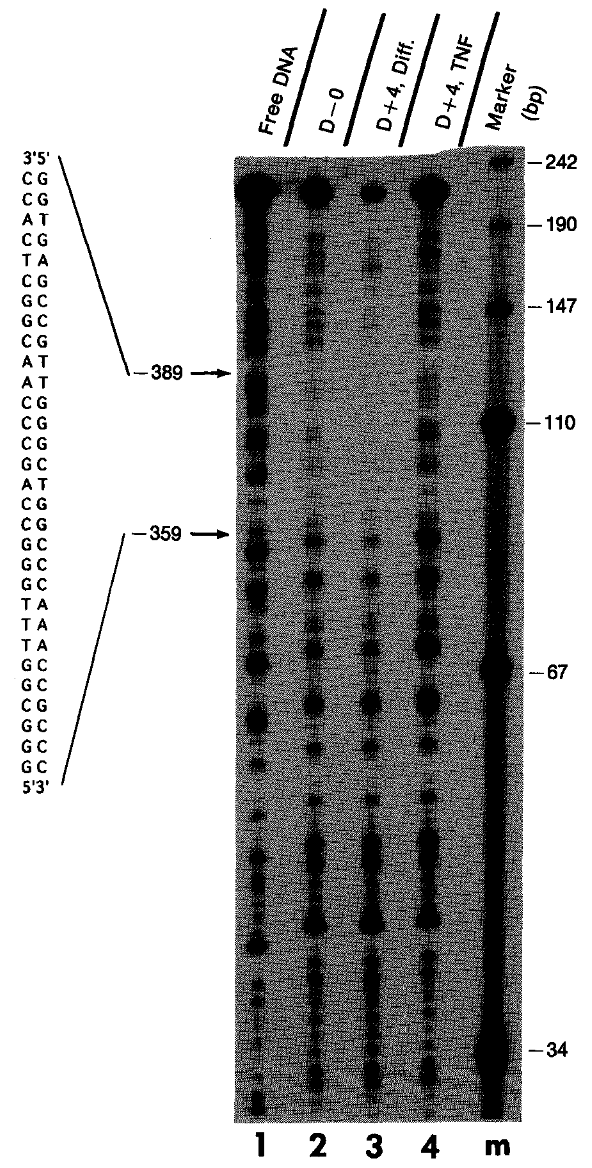

FIG. 4. DNase I footprinting analysis of DNA–protein interactions at ACC promoter II. Footprinting analysis was performed with the −434 to −285 fragment of pPII–TK2 construct labelled in the non-sense strand (lanes 1 to 4). The probes were incubated with nuclear extracts (40 μg) from 30A5 preadipocytes at confluency (lane 2) or the fully differentiated adipocytes at day +4 (lane 3) or TNF treated cells at day +4 (lane 4). These binding mixtures were then subjected to DNase I (0.5 U/2 μl for lane 1, 5U/2 μl for lanes 2 through 4) treatment for 2 min at room temperature as described in Materials and Methods. Lane 1 indicates the free probe DNA. Lane m represents the molecular weight markers ([$\gamma$-$^{32}$P] end labelled HpaII fragments of pUC 19) whose lengths in nucleotides are indicated to the right. The protected regions are indicated by arrows and the numbers correspond to the position of the nucleotides relative to the transcription initiation site. The protected sequences of the ACC PII promoter are shown in the figure.

these experiments show that the effect of TNF is mediated by a *cis* element in the −434/−285 region.

*Nuclear proteins binding to the TNF responsive element:* The −434 to −285 region and its relationship to TNF action on ACC promoter II was further characterized by determining whether specific nuclear proteins interacted with these sequences. To do this, DNase I footprinting analysis was performed using DNA fragment −434 to −285 of promoter II which was labelled at the 5′ end of the non-sense strand, and nuclear extracts prepared from cells with or without TNF treatment (Fig. 4). This analysis reveals that a 30 base pair from −389 to −359 of the non-sense strand was protected in the presence of the nuclear extracts from the differentiated cells (Fig. 4, lane 3) whereas the nuclear extracts from the TNF treated cells did not protect the same region (Fig. 4, lane 4). Nuclear extracts from the pre-adipocytes also did not protect the region, as shown in Fig. 4, lane 2. In order to further characterize the nuclear proteins bound to the TNF responsive region spanning −389 to −359, the 30 base pair sequence from −389 to −359 was synthesized and the DNA mobility shift analyses were performed (Figs 5 and 6). Four DNA bands were shifted in a sequence specific manner in the competition experiment (Fig. 5) and the formation of one of the four bands decreased when the cells were treated with TNF, as shown in Fig. 6, lane 2. In a separate experiment, it was determined that bands 1 and 2 are due to the binding of Sp1 to the 30-mer probe (data not shown).

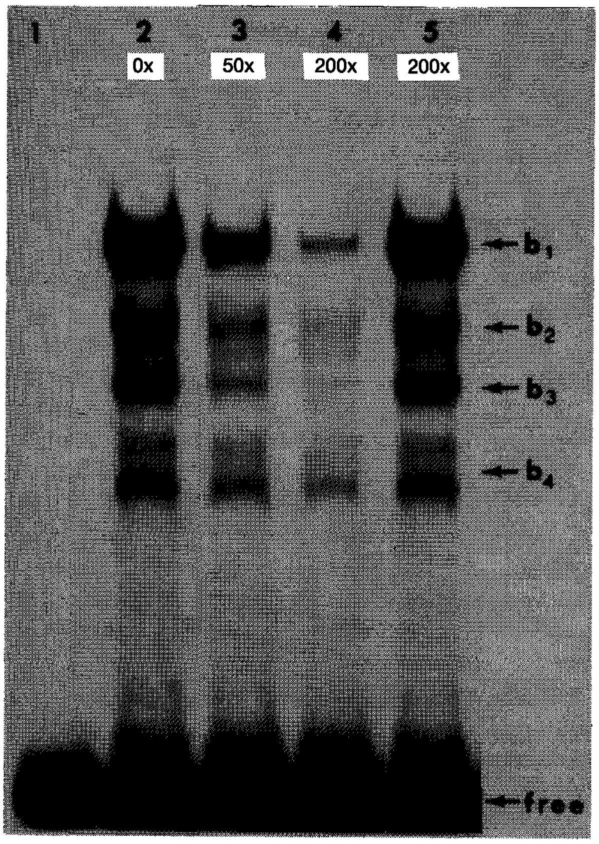

FIG. 5. DNA mobility shift binding profile. The ACC promoter II fragment spanning from −389 to −359 was end labelled and used for the binding assays as described in Materials and Methods. The nuclear extracts from differentiated 30A5 cells (D+4) were added to the binding reactions. The molar excess of unlabelled competitor DNA in the binding mixtures is indicated above each lane. Lane 1, the $^{32}P$ labelled DNA probe; lanes 2 to 5, the labelled 30-mer probe DNA in the presence of 30A5 nuclear extracts; lanes 3 and 4, in the presence of different amounts of the same unlabelled 30-mer; lane 5, in the presence of a nonspecific sequence (30-mer) of the ACC promoter I fragment spanning from −291 to −207.

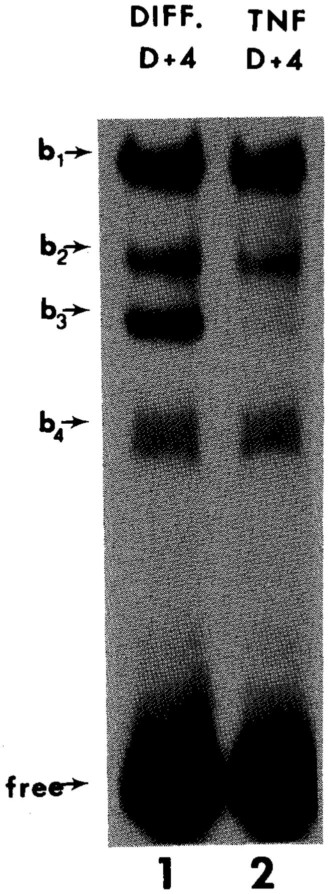

FIG. 6. DNA mobility retardation profile by nuclear proteins. End labelled 30-mer probe (−389 to −359), (0.2 ng), was incubated in the presence of nuclear proteins (10 μg) on ice for 30 min. The mobility of the retarded probe DNA was analysed through a 4% polyacrylamide gel as described in Materials and Methods. Lanes 1 and 2, labelled DNA (−389/−359) in the presence of the nuclear proteins from differentiated 30A5 cells (D+4) and 30A5 cells treated with TNF for the same period of time (D+4).

*Effect of −389/−359 on TNF responsiveness:* To further establish that the sequences in the −389/−359 region are responsible for binding the nuclear proteins whose binding activities were diminished by the treatment of cells with TNF, the effect of the addition of the −389/−359 fragment to pPII–CAT4 on the expression of CAT activity was examined (Fig. 7). The plasmid containing the 30 base pairs was prepared as follows. Plasmid pPII–CAT4 was linearized with SacI, blunt-ended by T4 DNA polymerase, and then the −389/−359 fragment was inserted in the site to create pPII–CAT8 (Fig. 1A). TNF inhibited the expression of the CAT gene in plasmid pPII–CAT2 by about 70%. However, CAT activity in plasmid pPII–CAT4 which does not contain the −434/−285 region was not inhibited by TNF. However, when the 30 bp fragment was placed in the pPII–CAT4 (pPII–CAT8), TNF responsiveness was reversed to an inhibition level of about 70%. These data suggest that the sequences within −389/−359 are responsible for conferring TNF responsiveness on the ACC gene. The sequence of this 30 bp region is GGTGAGCCGTTGGGCTG-GCCCAAACCGCCC (Fig. 8).

*Effect of the mouse TNF responsive region on TNF mediated transcription suppression:* In a separate experiment, we identified a TNF responsive sequence in the mouse gene that is homologous to the rat gene. The mouse ACC promoter sequence spanning the −370 to −340 region is highly homologous to the 30-mer sequence of the rat gene (Fig. 8). To examine whether or not the mouse sequence (−370 to −340) also shows TNF mediated transcription suppression, the synthesized mouse 30-mer was inserted in pPII–CAT4 vector which was linearized with *Sac*I and blunt-ended to construct pPII–CAT4 mTNF No. 3 (right orientation) and No. 22 (opposite orientation). We examined the effects of TNF on two constructs with the 30-mer in either direction as shown in Fig. 9. In comparison to pPII–CAT4 as a negative control and pPII–CAT 2 as a positive control, TNF inhibited the expression of the CAT gene by about 60% with both the mouse constructs. These results show that the mouse

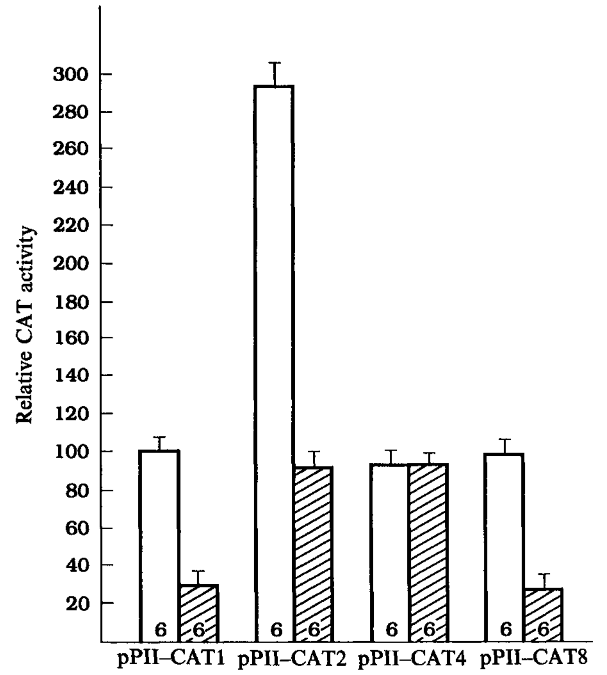

FIG. 7. Effect of the 30-mer *cis* element (−389/−359) on TNF action. The constructed plasmid (pPII–CAT8, Fig. 1A) containing the fragment (−389/−359) at the position −285 (pPII–CAT4) was transfected into 80–90% confluent 30A5 cells. The cells were harvested for CAT assay after 60 h of incubation in the presence or absence of TNF. The CAT activity obtained from pPII–CAT1 which was included in each experiment was set as 100%. The number of plates used to obtain data for each bar is indicated. Error bars indicate the range of three determinations. Key as Fig. 2.

```
Rat:    (-389)  GGTGAGCCGTTGGGCTGGCCCAAACCGCCC  (-359)
Mouse·  (-370)  GGTGGAGGTTGGCCGGCCCAAACCCGCCTA  (-340)
```

FIG. 8. TNF responsive sequences rat and mouse.

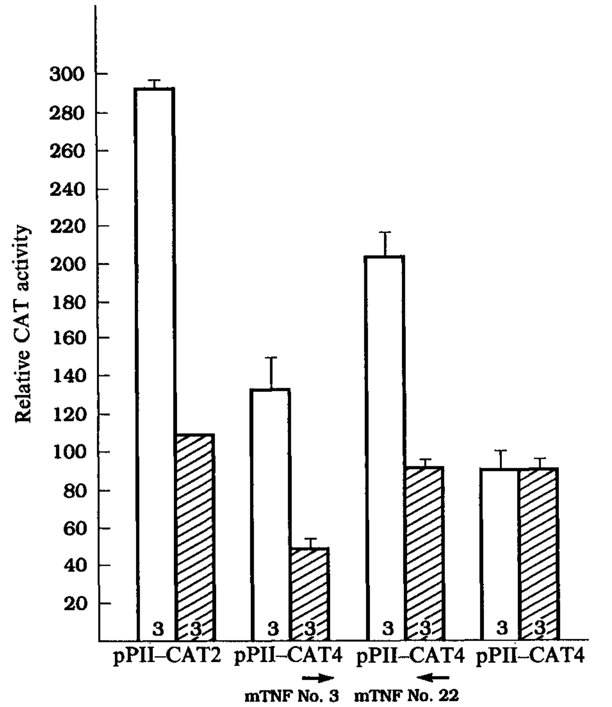

FIG. 9. Effect of the mouse TNF responsive region on expression of ACC–CAT construct. Two mouse constructs, pPII–CAT4 mTNF No. 3 and No. 22, were transfected into 80% confluent 30A5 preadipocytes together with pPII–CAT2 and pPII–CAT4. Sixty hours later, CAT activities in cell lysates were assayed. The CAT activity of pPII–CAT1 included in this experiment was taken as 100%. Error bars indicate the range of three determinations. Key as Fig. 2.

sequence also effectively mediates TNF effect on the gene expression in spite of minor differences in sequence from that of rat.

### Discussion

The effect of TNF on 30A5 preadipocytes has proved extremely useful for detailed study of ACC gene expression and thus, for elucidating the underlying molecular mechanisms for TNF action. Several investigators have studied the effect of TNF on lipogenic genes in various preadipocyte cell lines and in all cases TNF has been shown to inhibit the expression of this class of genes as determined by measuring their respective mRNA amounts.[4,20,21] In some cases, direct measurement of transcription rates by either labelling cellular RNA or nuclear run-off transcription assays reveals that the decrease in the amount of mRNA for the lipogenic gene under study is due to inhibition of gene transcription.[10,21] Such studies suggest that TNF response elements exist at the 5′ end of these lipogenic genes to confer TNF mediated inhibition of transcription. The authors provide evidence that such elements do exist in at least one lipogenic gene, i.e. the ACC gene, and these elements can confer TNF responsiveness to a non-lipogenic promoter.

Construction of 5′ deletion mutants of ACC promoter II placed upstream of the CAT gene revealed that the −434 to +62 region inhibited CAT activity approximately 70% upon TNF treatment in comparison to controls. Further deletions appeared to limit the TNF response as the −340 to +62 construct only suppressed CAT activity by about 30%. In many different experiments, we have observed that TNF can suppress about 30% of any gene construct irrespective of the nature of the promoters. These observations suggest that TNF action independent of the *cis* acting sequences in the promoters may still exist. These observations together with the results reported in this paper, suggest that the effect of TNF is mediated through multifaceted events. One of these is mediated through the *cis* acting elements in the promoter, in this case, ACC promoter II in combination with nuclear *trans* acting factors.

TNF treatment of 30A5 cells reduces the specific binding of nuclear proteins to the −389/−359 region of ACC promoter II. Whether the reduced binding is the result of a decrease in the amount of these factors, or some post-translational modification, is currently under study. The nature of these

*trans* acting factors is unknown; a careful search for consensus sequences of known transcription factors[22–24] does not reveal strong homologies. In addition, a homology search for the 5′ ends of other lipogenic genes also yields minimal homology. Thus, the transcription factors responsible for mediating the TNF responsiveness of ACC promoter II may be novel in both sequence specificity and/or regulatory properties.

Repression of a eukaryotic gene can occur through induction of repressor, or conformational changes in activator molecules, i.e. glucocorticoid receptor molecules bound to the glucocorticoid responsive element in the promoter region. During the action of TNF, it is found that the nuclear binding proteins on the 30-mer region diminished either through modification or a decrease in their amount. A simply hypothesis is that the binding of the nuclear protein is essential for activation of the ACC gene, the promoter, and that a decrease in binding activity results in repression. However, such regulatory mechanisms are rare. Recently, Brennan *et al.*[25] reported that serum, which inhibits myoblast differentiation, and thus various associated aspects of phenotypic gene expression, diminished the *in vitro* DNA binding activity of myogenin, suggesting that serum inhibition of myogenesis is due to the attenuation of the DNA binding activity of myogenin.

The underlying mechanism(s) for the decrease in the binding activities in the present case, or the effect of serum on myogenesis,[25] is not known, although a mechanism such as covalent phosphorylation can easily be envisaged.

### References

1. Beutler B, Mahoney J, Le Trang N, Pekala P, Cerami A. Purification of cachectin, a lipoprotein lipase-suppressing hormone secreted from endotoxin induced RAW 264.7 cells. *J Exp Med* 1985; **161**: 984–995.
2. Oliff A, Defeo-Jones D, Boyer M, *et al.* Tumours secreting human TNF/cachectin induce cachexia in mice. *Cell* 1987; **50**: 55–63.
3. Oliff A. The role of tumour necrosis factor (cachectin) in cachexia. *Cell* 1988; **54**: 141–142.
4. Min HY, Spiegelman BM. Adipsin, the adipocyte serine protease: gene structure and control of expression by tumour necrosis factor. *Nucl Acids Res* 1986; **14**: 8879–8892.
5. Pape ME, Kim KH. Effect of tumour necrosis factor on acetyl-CoA carboxylase gene expression and pre-adipocyte differentiation. *Mol Endocrinol* 1988; 2: 395–403.
6. Patton JS, Shepard HM, Wilking H, *et al.* Interferons and tumour necrosis factors have similar catabolic effects on 3T3-L1 cells. *Proc Natl Acad Sci USA* 1986; **83**: 8313–8317.
7. Torti FM, Beutler B, Cerami A, Ringhold GM. A macrophage factor inhibits adipocyte gene expression: an *in vitro* model of cachexia. *Science* 1985; **229**: 867–869.
8. Torti FM, Torti SV, Larrick JW, Ringhold GM. Modulation of adipocyte differentiation by tumor necrosis factor and transforming growth factor beta. *J Cell Biol* 1989; **108**: 1105–1113.
9. Lane MD, Moss J, Palakis SE. Acetyl-coenzyme A carboxylase. *Curr Top Cell Regul* 1974; **8**: 139–195.
10. Pape ME, Kim KH. Transcriptional regulatiön of acetyl coenzyme A carboxylase gene expression by tumour necrosis factor in 30A5 preadipocytes. *Mol Cell Biol* 1989; **9**: 974–982.
11. Luo X, Park K, Lopez-Casillas F, Kim KH. Structural features of the acetyl-CoA carboxylase gene: mechanisms for the generation of mRNAs with 5′ end heterogeneity. *Proc Natl Acad Sci USA* 1989; **86**: 4042–4046.
12. Luo X, Kim KH. An enhancer element in the house-keeping promoter for acetyl-CoA carboxylase gene. *Nucl Acids Res* 1990; **18**: 3249–3254.
13. Pape ME. Ph.D. thesis submitted to Purdue University, West Lafayette, IN 1989.
14. Park K, Kim KH. Regulation of acetyl-CoA carboxylase gene expression. *J Biol Chem* 1991; **266**: 12249–12256.
15. Maniatis T, Fritsch EF, Sambrook J. *Molecular Cloning: A Laboratory Manual.* Cold Spring Harbor Laboratory. Cold Spring Harbor, NY 1982.
16. Gorman CM, Moffat LF, Howard BH. Recombinant genomes which express chloramphenicol acetyltransferase in mammalian cells. *Mol Cell Biol* 1982; **2**: 1044–1051.
17. Crabb DW, Dixon JE. A method for increasing the sensitivity of chloramphenicol acetyltransferase assays in extracts of transfected cultured cells. *Anal Biochem* 1987; **163**: 88–92.
18. Fregien N, Davidson N. Activating elements in the promoter region of the chicken beta-actin gene. *Gene* 1986; **48**: 1–11.
19. Dignam JD, Lebovitz RM, Roeder RG. Accurate transcription initiation by RNA polymerase 2 in a soluble extract from isolated mammalian nuclei. *Nucl Acids Res* 1983; **11**: 1475–1489.
20. Dobson DE, Groves DL, Spiegelman BM. Nucleotide sequence and hormonal regulation of mouse glycerophosphate dehydrogenase mRNA during adipocyte and muscle cell differentiation. *J Biol Chem* 1987; **262**: 1804–1809.
21. Zechner R, Newman TC, Sherry B, Cerami A, Breslow JL. Recombinant human cachectin/tumour necrosis factor but not interleukin-1 α down-regulates lipoprotein lipase gene expression at the transcriptional level in mouse 3T3–L1 adipocytes. *Mol Cell Biol* 1988; **8**: 2394–2401.
22. Reynolds GA, Basee SK, Ooborne TF, *et al.* HMG–CoA reductase: A negatively regulated gene with unusual promoter and 5′ untranslated region. *Cell* 1984; **38**: 275–285.
23. Singer-Sam J, Keith DH, Tani K, *et al.* Sequence of the promoter region of the gene for human X-linked 3-phosphoglycerate kinase. *Gene* 1984; **32**: 409–417.
24. Wingender E. Compilation of transcription regulating proteins. *Nucl Acids Res* 1988; **16**: 1879–1902.
25. Brennan TJ, Edmonson DG, Li L, Olson EN. Transforming growth factor β represses the actions of myogenin through a mechanism independent of DNA binding. *Proc Natl Acad Sci USA* 1991; **88**: 3822–3826.

ACKNOWLEDGEMENTS. This work was supported by Public Health Service grant CA 46882 from the National Institutes of Health. This is journal paper number 12651 from the Purdue University Agriculture Experiment Station.

**Received 25 March 1993; accepted after revision 20 April 1993**
